# Development of a digital learning program for physiotherapists to enhance clinical implementation of aerobic exercise in stroke rehabilitation

**DOI:** 10.1186/s40945-021-00110-5

**Published:** 2021-06-17

**Authors:** Marianne Thornton, Jennifer Harris, Krista Breithaupt, Tracey Dyks, Hillel Finestone, Marilyn MacKay-Lyons

**Affiliations:** 1 Champlain Regional Stroke Network, 2221 Carnegie St, Ottawa, K1G 2V4 Ontario, Canada; 2 Ottawa Heart Institute, 40 Ruskin Avenue, Ottawa, K1Y 4W7 Ontario, Canada; 3 University of Ottawa, c/o 174 Meandering Brook Drive, ON K1T O3C Ottawa, Canada; 4 The Ottawa Hospital, 501 Smyth Road, Ottawa, K1H 8L6 Ontario, Canada; 5 Elisabeth Bruyère Hospital, 43 Bruyère St, ON K1N 5C8 Ottawa, Canada; 6 Dalhousie University, Forest Building, 6299 South St, NS B3H 4R2 Halifax, Canada

**Keywords:** Stroke rehabilitation, Aerobic exercise, eLearning, Evidence-based practice, Knowledge translation

## Abstract

**Background:**

This paper describes the initial development process of an eLearning continuing professional education program primarily for post-licensure physiotherapists –“Electronic Aerobic Exercise Recommendations to Optimize Best Practices in Care after Stroke” (eAEROBICS). Our objective was to develop an evidence-based, clinically relevant, user-friendly eLearning program for online delivery tailored to facilitate prescription of aerobic exercise post-stroke by physiotherapists. The Demand Driven Learning Model guided curriculum design, delivery, and evaluation. Based on previously identified gaps in physiotherapists’ knowledge of aerobic exercise, four learning modules were developed and delivered using an eLearning platform to maximize cost-effectiveness and flexibility. Five physiotherapists volunteered to pilot eAEROBICS, providing preliminary feedback on strengths and suggestions for improvement.

**Results:**

Theoretical information and clinical applications addressed the learning objectives of each module in a logical manner. All technical or administrative issues encountered during program delivery were addressed. The feedback from the pilot end-users informed modifications to the eAEROBICS program.

**Conclusions:**

Processes used in developing eAEROBICS have the potential to serve as a model of electronic continuing professional education for other areas of physiotherapy practice. Further investigation of end-user perspectives and clinical impact of the program is warranted to determine the overall effectiveness of the program.

## Background

According to the most recent available information, there are currently over 400,000 people living with the effects of stroke in Canada [1–3]. There is a 20–40% chance [depending on gender and time post stroke) that someone who suffers a stroke will have a second stroke within two years [4, 5] The escalating cost of healthcare [5] has motivated growing emphasis on efficiencies in healthcare delivery [6], driving a shift in service provision from in-patient to out-patient, community-based care [7]. Traditionally, it has taken an average of 17 years to translate research findings into practice [8–10]. From the perspective of physiotherapists, limited professional time and access to evidence-based knowledge have contributed to prolonging the process of implementing changes in practice [11]. Compelling evidence exists for numerous benefits of aerobic exercise (AE) post stroke, most importantly in management of cardiovascular risk factors [12]. Recently, recommendations to guide clinical utilization of AE — Aerobic Exercise Recommendations to Optimize Best Practice in Care after Stroke (AEROBICS) [13] — have been integrated into Canadian Best Practices Recommendations for Stroke Care [14] and American Heart Association’s Physical Activity and Exercise Recommendations for Stroke Survivors [15]. Prior to this time, most stroke survivors remained quite physically inactive in hospital or at home, and even during physiotherapy sessions. [16, 17]. Despite widespread low cardiorespiratory fitness among stroke survivors, AE has continued to be underutilized during stroke rehabilitation [18, 19]. One factor mediating the disparity between practice recommendations and actual practice is physiotherapists’ lack of self-efficacy (confidence in executing a task) related to screening for, and prescribing, AE for people post stroke [19, 20].

Knowledge translation (KT) strategies, including eLearning, have been shown to influence clinician self-efficacy and practice behaviors but the effects have been modest at best [21–23] The impact of eLearning on clinical practice and patient outcomes remains under-investigated [24]. Several impediments exist that limit the changes in the culture of practice required to embed evidence-based knowledge into practice. Canadian physiotherapists are dispersed across a vast geographic area, [25, 26] and therefore it can be challenging to organize face-to-face workshops or seminars, which are the most commonly used KT strategy in rehabilitation [22]. As well, passive modes of continuing professional education (CPE) such as the distribution of printed educational material have been found to be largely ineffective [21, 27] Fortunately, advances in eLearning theory and technology have made it possible for healthcare professionals to access best practice education more conveniently, without the time and cost of travel to a centralized campus [28]. As a consequence, there has been a gradual transition from ‘point in time’ learning toward asynchronous, more flexible delivery approaches, including eLearning [29]. Digital technologies offer the learner control over learning sequence, pace of learning and time allotment, which allows tailoring experiences to meet personal learning objectives [30]. However, there is limited knowledge of perceived usability among health professionals of new knowledge exchange strategies for CPE [30, 31].

Recognizing the lack of self-efficacy regarding AE screening and prescription among physiotherapists in stroke rehabilitation, and the need for ready access to evidence-based, AE-related knowledge, we developed a novel CPE program entitled eAEROBICS. Five stages in the development and evaluation were identified by the eAEROBICS team: Stage 1: Design of a curriculum to prepare physiotherapists for safe and effective implementation of aerobic training in stroke rehabilitation; Stage 2: Delivery of the curriculum in an efficient, interactive and cost-effective manner; Stage 3: Preliminary evaluation of end-user feedback on the curriculum, delivery, and services of the eAEROBICS program; Stage 4: A more comprehensive evaluation of end-user perspectives of the program, using the four domains of the Kirkpatrick model of learning evaluation – reaction to learning, learning (knowledge, skills, attitude, and self-efficacy), clinician behavior (recommendation adherence), and patient results [32]; Stage 5: Evaluation of the impact of eAEROBICS with clinical data.

Given the importance of documenting the processes used to enhance clinical uptake of recommendations, [33, 34] this paper focuses on Stages 1–3. The overall objective is to develop an evidence-based, clinically relevant eLearning program for online delivery tailored to facilitate prescription of exercise post-stroke by physiotherapists. Details are presented of the development and the findings of a preliminary evaluation of the eAEROBICS development. Results of Stages 4 (comprehensive end-user evaluation) and 5 (impact evaluation) will be addressed in a subsequent paper.

## Methods

Important barriers to AE implementation in practice include lack of access to relevant knowledge and low therapist confidence [11, 19, 20]. We sought to address these obstacles through online education including practical cases that represented real-life practice scenarios. The focus of Stages 1 and 2 was on development of the eAEROBICS Program and for Stage 3 on preliminary evaluation of the program.

**Fig. 1:**
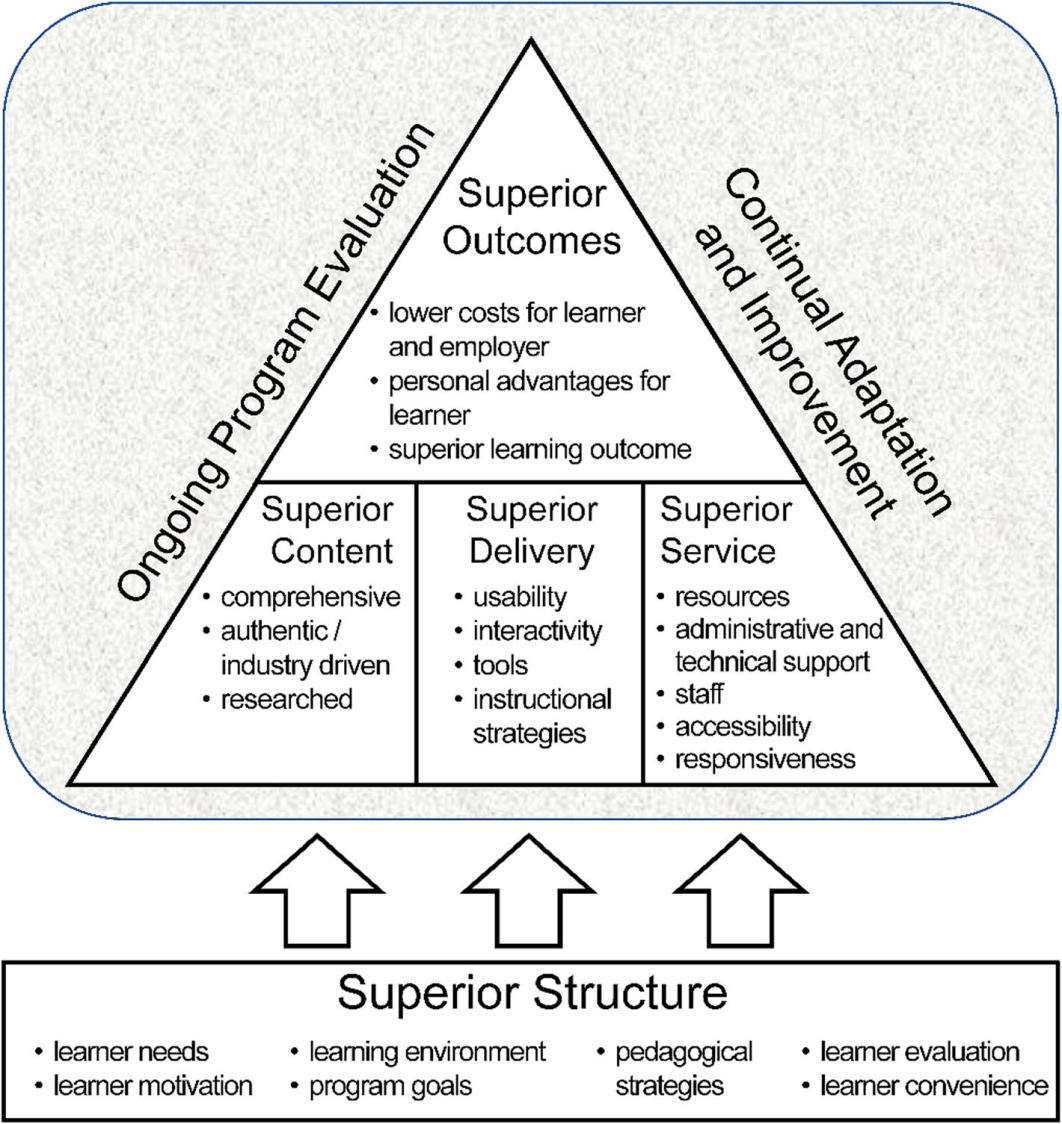
Demand Driven Learning Model. The DDLM theoretical framework used in development of eAEROBICS is shown in Fig. 1. The DDLM combines adult learning principles with eLearning technologies based on best educational practices. The model shows the relationship of the five critical dimensions of online learning—structure, content, delivery, service, and outcomes

**Fig. 2:**
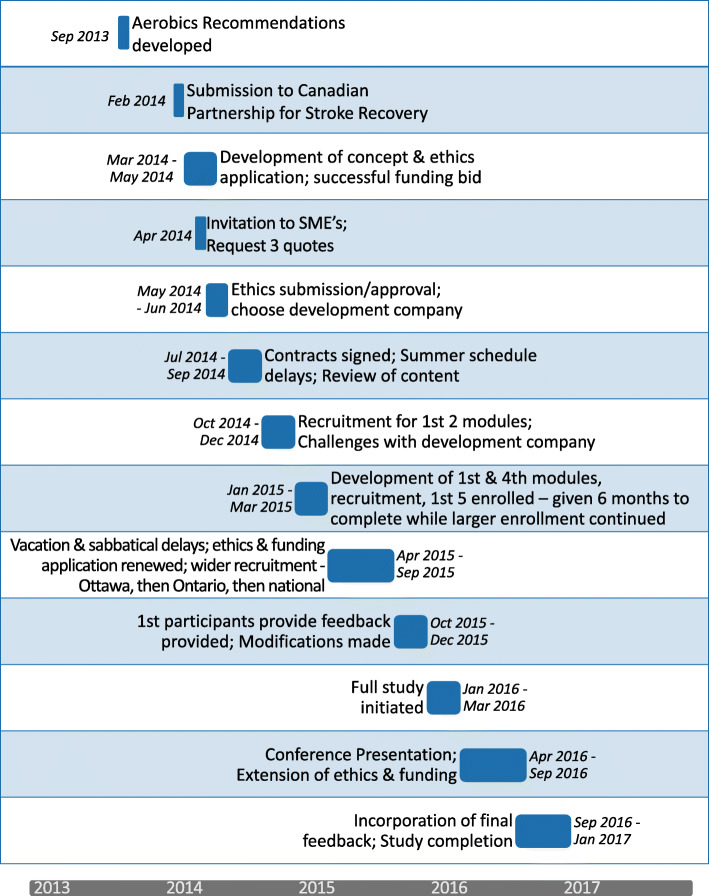
eAEROBICS Development Timeline. Timeline for eAEROBICS development showing the major development points during the process

Development of the eAEROBICS Program: We chose the Demand Driven Learning Model (DDLM) [28] as the theoretical framework to ground development of the eAEROBICS program. The DDLM combines adult learning principles with eLearning technologies based on best educational practices, and is designed to specifically address the “needs, interests, and lifestyle demands of the working adult learner” [35]. The model encompasses 5 critical dimensions of online learning—structure, content, delivery, service, and outcomes (Fig. 1) [36]. The methods used in Stages 1 and 2 are described below in terms of these dimensions. The timeline for the initial development of eAEROBICS, from July 2014 to January 2017, is illustrated in Fig. 2.

### Stage 1 -design of the eAEROBICS curriculum

The first two critical dimensions of the DDLM framework – structure and content – were of foremost concern in designing the eAEROBICS curriculum.

#### Structure

To establish a foundational structure for the curriculum of the eAEROBICS program, we turned to previous literature reporting gaps in knowledge of practicing physiotherapists that could adversely affect clinical implementation of AE in stroke rehabilitation [19, 37]. Among these gaps were: (i) uncertainty of how to determine the safety of patients to engage in AE, (ii) lack of confidence in designing an appropriate AE program, (iii) inadequate knowledge of strategies to encourage the changes in behaviour needed to engage patients in AE, (iv) limitations in knowledge of underlying physiological principles related to AE. A Clinician’s Guide called ‘Aerobic Exercise After Stroke’ [38] was created, which provided another way to package the Recommendations and assist with knowledge translation. This resource was not able to provide practical examples to link the recommendations with practice. Thus, we chose a modular structure for the curriculum, to build in critical thinking, consisting of 4 modules: (i) Self-Management and Behaviour Change, (ii) Fundamentals of Exercise Physiology, (iii) Screening for Safe Participation in AE after Stroke, and (iv) Prescription of AE for People Post Stroke.

#### Content

To optimize knowledge translation the eAEROBICS curriculum was designed to be learner-centered, evidence-based, and clinically relevant. Specific learning objectives were identified for each module. Subject matter experts (SMEs) – a physiotherapist with expertise in stroke neurotherapeutics, focusing on aerobic exercise and its application to aerobic capacity to reduce morbidity; a physiotherapist with expertise in exercise physiology; and a health psychologist with expert knowledge in health behaviour change associated with cardiovascular risk – were consulted to develop content relevant to the learning objectives. Each of these SMEs had doctoral degrees in the subject matter. Information presented in each module was comprehensive yet concise, and grounded in accessible, empirical evidence from the evidence-based AEROBICS recommendations [13] and other relevant literature (e.g. [39–42]. The content was organized in a logical manner with theoretical information providing a foundation for the clinical application that followed. Each module was introduced by video recording of the SME summarizing the content and learning objectives. Learning tasks were moderately complex, simulating workplace situations. Optional, more in-depth, learning opportunities were included throughout the modules with direct links to relevant literature, practice guidelines, and additional case applications.

The SMEs were consulted on an ongoing basis throughout the curriculum design, delivery, and evaluation processes. Their feedback was utilized to ensure that each content area was presented authentically, in a clinically relevant manner, and was reflective of key elements of the DDLM Framework.

### Stage 2 -delivery of the eAEROBICS curriculum

The third and fourth critical dimensions of the DDLM framework – delivery and service – were important considerations in this stage of development of our eLearning CPE program.

#### Delivery

The mode of delivery of an eLearning curriculum must be carefully selected to ensure functionality and interactivity [36]. A stand-alone, html-based mode was chosen for this online curriculum allowing for low-cost implementation and use of standard technology, both of which facilitated uptake by our primary stakeholders (physiotherapists practicing in stroke rehabilitation). An iterative process was used with our application architect and programmers, and standard web conventions were applied. The goal was to afford ease of navigation and intuitive interfaces designed for learners to enhance engagement and increase motivation to complete each module. To enhance comprehension the text was augmented in several ways: voice-over commentary, key messages summarizing content of each module, and visually engaging illustrations. In addition, clinical scenarios and case studies with video footage were used to integrate theory and clinical practice. Flexibility was enhanced by ensuring segments of modules could be completed in 15 min [43], with the participant able to bookmark their current learning activity. As well, the Participant was able to flag material particularly relevant to the individual’s clinical practice.

Interactivity within eAEROBICS was regarded as key to both self-assessment and tailoring learning experiences to the needs of the individual end-user. To achieve these objectives, reflective questions and short quizzes using multiple choice, true or false, and matching formats, developed by the SMEs, were interspersed throughout the program, with feedback provided on the learner’s performance on the quizzes.

#### Service

Provision of administrative and technical assistance is fundamental in eLearning environments to ensure accessibility and responsiveness [28, 44, 45]. The eAEROBICS program was designed to facilitate uncomplicated access to the learning tool, smooth navigation of modules, timely feedback to quiz results, and technical support to address requests for assistance.

### Stage 3 - preliminary evaluation of the eAEROBICS program

The fifth and final critical dimension of the DDLM framework was the focus of Stage 3. Evaluation by end-users is a critical component in the on-going development of eLearning modules. An iterative process is recommended in order to improve the program over the course of development [28, 46, 47]. Thus, a pilot evaluation of eAEROBICS was planned from the outset to obtain early feedback from end-users of the program, with further program evaluation to follow in Stages 4 and 5. Approval of the enrolment procedure and study protocol for Stages 1–3 was received from the Ottawa Health Science Network Research Ethics Board (protocol #20140367-01H). Given the preliminary nature of the Stage 3 evaluation and the more comprehensive Stage 4 evaluation, we sought to recruit a sample of 4–6 physiotherapists with a minimum of five years of clinical experience in stroke rehabilitation in Canada, through email advertisements distributed to the Canadian universities, the Canadian Physiotherapy Association and health care facilities. Each participant provided written informed consent.

Upon completion of each module, email feedback was solicited from each participant to the open-ended questions of ‘what worked’ and ‘what could be improved’ in terms of (i) content and structure of the curriculum related to each learning objective, (ii) delivery of the program, and (iii) services available to aid navigation of the module. After completion of all four modules, one of the researchers interviewed each participant by phone using a format similar to that used in the email feedback. The interviewer took notes of each participant’s responses, which were later entered into a spreadsheet. One researcher (MT) coded the responses by participant number (1-5) and grouped the responses under two main headings – strengths and areas for improvement – and 3 subheadings – content, delivery, and service. A second researcher (MM-L) checked the groupings. Disagreements were resolved by discussion. In the Results section that follows, the feedback from the participants is summarized.

## Results

Five female physiotherapists volunteered to participate in the pilot evaluation of the eAEROBICS program. Three had a Bachelor of Science degree in Physiotherapy and 2 had a Master of Science degree in Physiotherapy. Two worked in the area of general rehabilitation providing mostly one-on-one therapy and 3 worked in community rehabilitation providing in home therapy. All had over 5 years of clinical experience treating patients post stroke. All treated patients that were predominantly stroke but saw a mix of neurology cases. Two of the therapists worked with under 5 stroke patients per week, 3 worked with 5–10 patients per week. All worked in medium to large sized cities. The total running time of the individual modules were:

- Module one (Behaviour Change) 21 min (m) 50 sec (s)
- Module two (Exercise Physiology) 26 m 50 s
- Module three (Screening for AE) 27 m 02 s plus 2x10 m case studies
- Module four (Prescription of AE) 27 m 40 s plus 2x12 m case studies totalling 150 m 47 s (2 h 30 m 47 s)

Actual time spent on each module by participants is difficult to determine, as they could leave and return to the module without limit. The goal of 30 min total to complete each module was an important component to making it accessible to busy therapists.

In terms of delivery and service, no insurmountable technical or administrative issues were encountered in the delivery of the eAEROBICS program. Response time to email inquiries from participants seeking assistance was within 24 h, on average. Each participant provided feedback on every module. The DDLM framework was used to categorize perceived strengths and suggestions for improvement into three themes -content, delivery, and service. Table 1 summarizes the feedback.

**Table 1:** Pilot study participants input regarding the strengths and areas for improvement of the eAEROBICS program categorized into 3 dimensions of the Demand Driven Learning Model

Content	Delivery	Service
**Strengths of the eAEROBICS Program**		
Participation in this pilot has helped me to increase the participation of some stroke patients in aerobic exercises. *002*	I liked that I was able to move back in the slides to review information at any time, including during a quiz. *001*	Providing immediate feedback on responses to quiz questions aided learning. *005*
Tables summarizing the content were excellent. *004*	Slides were clear and easy to read. *005*	I liked the links throughout the module to various resources that supplemented the information in the slides. *005*
Module content was relevant to practice. Second module was a good review *003*	Quizzes to assess understanding were beneficial *001*	
I like the attention given to behaviour modification.” …behaviour modification section was really helpful, as this is a common issue we deal with in practice. *004*	Introductory videos and audio from the subject matter experts were helpful learning tools. *002*	
Section on motivational interviewing was very interesting! *003*	Video footage of cases made learning more life-like and applicable. *005*	
The demo video was helpful *001*	The graphics are used effectively to highlight key information *005*	
Case studies were effective at pulling the information together and reviewing the tools and information *005*	I found the course content well laid out and easy to follow. *005*	
**Areas for improvement of the eAEROBICS Program**		
Practical issues such as how to access exercise stress tests should be addressed. *002*	The process of how to navigate through the modules should be made more evident. *004*	A paper manual as a reference guide would be a useful addition. *004*
Additional resources (e.g., workshops, hands-on practical sessions) would help to reinforce the content of eAEROBICS. *004*	A progress bar should be added to indicate how far along the learner is in terms of module completion. *003*	There was a time delay in receiving responses to email inquiries. *005*
I found the introductory piece very lengthy. As an experienced clinician, the behaviour module was not useful. *004*	A pop-up option should be added to provide formulae to assist in answering quiz questions that involve calculations. *003*	More technical support is needed to respond to requests for assistance. *003*
	Volume of various video clips was not consistent. *005*	Missed having a mechanism to ask questions *004*
The formulas for HRR were a little more complex and could have had more examples of calculation. *003*	Pace of narration (voiceover) was too slow at times. *005*	Is it possible just to get an email/ certificate or something specifying participation in your research that can then go into my portfolio? *004*

Although the participants were largely positive about the content and delivery of the modules, they provided several constructive suggestions for improvements, including: (i) adding a program navigation guide in the introduction to the program, (ii) reducing the content in the introductory section, (iii) adding a progress bar to mark the percentage of the module completed, (iv) inserting on-screen buttons to allow feedback and request technical support, (v) re-taping the voiceover, (vi) including additional learning resources such as references and downloadable documents, and (vii) providing of program completion certificates, which could be used for CPE credits. Each of the recommendations has been incorporated in a revised eAEROBICS program, which is currently being evaluated using a larger sample (Stage 4).

## Discussion

Translating new research and guidelines into practice is an essential aspect of health care services [48]. eAEROBICS is unique as a CPE program in that it was developed by, and delivered to, practicing physiotherapists. Although much of the content of eAEROBICS had been available through traditional venues, eAEROBICS was initiated in response to a perceived need for more post-entry level education regarding the clinical implementation of AE in stroke rehabilitation [19].

Learning strategies must be carefully selected during the planning and development of CPE programs [49] The DDLM was selected as a framework to optimise the quality of the curriculum. This framework guided decision-making from conceptualization to the launch of the program, resulting in a clear structure of curricular content. Factors identified as important to adult learners – interactivity, flexibility, and accommodation of various learning styles and motivations [44, 50] – were foremost considerations. To enhance learners’ motivation to complete the eAEROBICS modules, we followed the advice of Gustafson and colleagues [51] to select content that is relevant, accessible, and meaningful to end-users. We also prioritized comprehensiveness and authenticity, which previously were shown to be ingredients of high-quality content [46]

To foster life-long learning, CPE programs should encourage learner involvement and provide meaningful educational experiences [52]. We embedded within each module strategies such as setting clear learning objectives, offering relevant type and amount of content, and evaluating learner knowledge. Problem-based reflective thinking was encouraged through interactive case studies with supporting videos that were applicable to different practice settings. Quizzes provided opportunities for self-assessment of the content, with immediate feedback on performance provided. In addition, appropriate information was given to reinforce correct answers, along with directing the participant back to relevant sections of the modules. Selecting an accessible, efficient, and cost-effective mode of delivery for CPE programs requires thoughtful planning. In Stage 2 of program development, we chose to use a digital learning platform because this approach is currently recognized as a practical and economical strategy for professional education development. The role of eLearning program developers has evolved from simply converters of information from written to digital format to facilitators of self-directed learning, whereby more emphasis is placed on the needs and experiences of learners than the instructor [30, 46, 53]. The flexibility afforded by asynchronous learning modes benefits busy clinicians who can individually adjust content viewing with respect to learning schedule, location, sequence, and pace. The time to complete each module [~ 30 min) and ability to stop and return was also a benefit to busy clinicians.

Providing opportunities for formative feedback has also been highlighted as a fundamental component of adult learning by theorists who develop CPE [28, 46, 47, 54, 55]. In Stage 3 of our project, the preliminary input of the small sample of end-users, shaped by their individual experiences and clinical contexts, offered invaluable insights. Their suggestions precipitated modifications to content, delivery, and service prior to formal launch of the eAEROBICS program. Importantly, they found the programme motivating and easy to navigate contrary to the findings in the literature stating the eLearning challenges included decreased learner motivation, time constraints, and lack of familiarity with digital learning formats [52, 56, 57].

### Limitations

The small sample size is a limitation, although it is acceptable given the pilot and qualitative nature of this phase of the project [58]. Also, it is important to note that response bias may have been introduced in the pilot evaluation of the eAEROBICS program because the sample was not completely representative of the population of Canadian neuro-physiotherapists. All participants were female, whereas in 2019, 72.4% of physiotherapists practicing in Canada were female [26]. Potential gender differences in learning styles as well as educational, clinical, and avocational experiences could influence perceptions of the eAEROBICS program. As a result, probabilistic sampling stratified by gender is currently being used in Stage 4 to ensure that external validity of the findings is not jeopardized.

### Future directions

Further investigation of the impact of eAEROBICS on physiotherapists’ learning as well as on actual implementation of AEROBICS recommendations in clinical practice is warranted.

## Conclusions

Development of eAEROBICS offers an example of the application of best practices in digital learning to create an eLearning program for physiotherapy post-licensure education. The eAEROBICS program was designed to be an effective way to facilitate evidence-based knowledge translation and encourage self-directed learning relevant to the clinical application of aerobic exercise. The processes used in curriculum development, delivery, and formative evaluation, guided by the DDLM framework, have the potential to serve as a model of electronic CPE for other areas of physiotherapy practice. Further investigation of the impact of eAEROBICS not only on perceptions of physiotherapists but also on actual implementation of AEROBICS recommendations in clinical practice is warranted.

## Data Availability

The datasets used and/or analysed during the current study are available from the corresponding author on reasonable request.
